# The use of evidence-based practices for the management of shoulder impingement syndrome among Indian physical therapists: a cross-sectional survey

**DOI:** 10.1590/bjpt-rbf.2014.0115

**Published:** 2015-10-06

**Authors:** Vandana Phadke, Meena Makhija, Harpreet Singh

**Affiliations:** 1 Clinical Research Department, Indian Spinal Injuries Center (ISIC), 110070, New Delhi, India; 2 Institute of Rehabilitation Sciences, (ISIC), 110070, New Delhi, India; 3 Department of Orthopedics, (ISIC), 110070, New Delhi, India

**Keywords:** shoulder pain, assessment, EBP

## Abstract

**Background:**

The understanding of the pathomechanics of shoulder impingement has evolved over the years. Likewise, assessment techniques and effective treatment strategies have also been developed. Physical therapists should keep up-to-date on the current evidence.

**Objective:**

This study explored the practices currently used by Indian physical therapists for the assessment and management of shoulder impingement syndrome (SIS).

**Method:**

Using an online questionnaire, therapists were asked to declare the causes, methods of assessment and their choices of physical therapy techniques for the management of SIS. The proportions of therapists using different techniques were analyzed descriptively, and comparisons across gender, experience level, and training were made. Data were analyzed to see if the choices of respondents compared with their responses for etiology.

**Results:**

A total of 211 responses were analyzed. Most respondents (>75%) believed that overuse and abnormal motion/posture are the most significant causes of SIS. However, fewer respondents reported assessing posture (60.2%) and dyskinesis, especially in women (24.2%). Ninety-four percent of the respondents reported using exercises, but exercise prescription was rather generic. Therapists additionally trained in the techniques of joint mobilization or taping declared using these techniques more frequently. The use of interferential therapy and ultrasound was reported by 89.5% and 98.4% of respondents, respectively

**Conclusion:**

Most therapists declared awareness of current recommended practices, but patient assessment, exercise prescription, and use of electrotherapy modalities were only partially based on current evidence. The study helps to identify gaps in current physical therapy approaches to SIS in India.

## Introduction

 Shoulder pain is a common clinical complaint in the outpatient departments of hospitals, ranking 3rd after low back pain and neck pain^1^. Shoulder impingement syndrome (SIS) is the most common cause of shoulder pain^2^. It commonly affects those exposed to overhead or repetitive activities^3^. 

The understanding of the pathomechanics of SIS and evidence-based guidelines to manage the condition has evolved over the years^4^. Neer^5^ claimed that the abnormal shape of the acromion causes mechanical compression of the rotator cuff tendons as they pass under the coracoacromial arch. Other studies^6^^,^ 7 postulated that, as the tendons are hypovascular near their insertion, repair from wear and tear associated with repetitive movement becomes increasingly difficult. Currently, SIS is considered a non-specific term that includes multiple diagnoses (inflammation or partial tears of rotator cuff tendons; involvement of the long head of biceps; bursal inflammation)^4^ and multiple subtypes (external or internal; subacromial or subcoracoid) and has a multifactorial etiology^3^. In addition to the intrinsic reasons, in recent years, efforts have been made towards analyzing the relationships between factors that may reduce the potential space for rotator cuff tendons in the subacromial space^8^^-^ 10 and thus trigger the pathological cascade. These include muscle strength imbalances, altered motor control, tightness of muscles/other soft tissue structures around the shoulder, or postural deviations3. This underscores the role of physical therapy in the assessment and management of SIS.

Recent research has shifted the focus of treatment towards a conservative and targeted approach^4^. Symptom alteration tests to identify patients that may benefit from the movement-based diagnosis and management have also been devised in the last two decades^11^^,^ 12. Recent evidence claims that emphasis to correct scapular and glenohumeral movement and associated muscle performance improves pain and functional scores in patients^13^^-^ 16.

Evidence-based practice has become the standard of care in today's world. Hence, clinicians need to stay up-to-date on the recent trends and changes. Numerous interventions are being used by physical therapists including exercises, manual therapy, taping, and electrotherapeutic modalities for the management of SIS^16^. If therapists are well acquainted with the most effective management strategies, the associated morbidity and overall socio-economic impact of the disorder may decrease.

Various review articles^13^^-^ 19 have outlined the relative effectiveness of various therapy techniques for the management of SIS. Although these studies prescribe the best possible ways to assess and treat a patient, few studies^20^^-^ 22 have documented the self-reported practices of therapists. Our study was designed to assess the knowledge, beliefs, and self-reported practices of Indian physical therapists in relation to causes, assessment, and management of SIS and to check if their responses suggest implementation of current evidence. The study also aimed to assess if the practices of therapists are consistent with the evidence regarding effectiveness of intervention changes in kinematics, motor control, and muscle function. Such studies are especially relevant in countries like India, where there is a lack of standard regulatory policies on academic and clinical physical therapy practices.

## Method

An online questionnaire in English was developed using the 'Survey Monkey' portal (Survey Monkey Inc., Palo Alto, CA, USA). The questionnaire included initial questions to obtain background information about the participant's age, gender, year of graduation, specialization, additional training, current employment setting, years of clinical experience, and the average number of shoulder pain cases treated in a year. The participants were then asked to rate the top three reasons that cause SIS and report what they examined clinically in the patients with this disorder.

A fictitious case of a patient diagnosed with SIS is presented below:

Case of shoulder impingement presented in the questionnaire: A 40-year old man who works as a schoolteacher complains of pain in his right shoulder for a period of 6 weeks. He is right side dominant and complains of pain whenever he tries to raise his arm overhead (numerical pain rating score 5/10). The pain started insidiously and he claims that he has never had shoulder problems or surgery before. The pain is now persistent, decreases temporarily with pain medications, and worsens while sleeping. The pain refers to the mid-arm, but there is no tingling or numbness on either side. There is no associated neck pain. There is no history of any trauma or unusual lifting of weights. He occasionally plays badminton and cricket over the weekend. There is no other significant medical or surgical history. Evaluation reveals that he has no limitation of active or passive range of motion at the shoulder joint, no apparent muscle weakness, and no shoulder instability, but shows signs of positive impingement.

Participants were subsequently provided with a choice of different physical therapy techniques. This list of options was made based on available literature, textbooks, and pilot testing. The questionnaire included the following: specific questions on choices of designing generalized versus individualized exercise programs; muscles to be strengthened (rotator cuff, scapular); exercises used (closed or open kinetic/endurance training/relaxation/neuromuscular control/use of feedback); soft tissue structures targeted for stretching; joints mobilized; use of taping; use of modalities (heat, cold, ultrasound, different currents); and activity modification instructions. The respondents were asked if they would use a particular technique for the above mentioned patient and what their level of confidence was in its effectiveness for the management of SIS. 

The respondents were then asked to choose from the options: 'Yes, I will use the technique. I am very confident/somewhat confident/not confident in the effectiveness of the technique' or 'No, I will not use the technique'. Additionally, in all the questions, the respondents were free to reply that they had no knowledge of a particular technique.

The questionnaire was designed in such a way that, if a respondent claimed to use a particular technique, ancillary questions were asked to get more details. It was emphasized to the participants that they should report what is actually used in their practice and not what ideally should be done.

The questionnaire underwent a pilot test with 10 therapists with adequate representation from different specialization fields and levels of experience. In this test phase, the participants were asked to give feedback concerning time taken, content, understandability, clarity, order, interest, and appropriateness. The changes suggested by the reviewers and later incorporated in the survey included adding questions regarding assessment of scapular motion in females and males separately and excluding a few open-ended questions. Data from pilot testing were not included in the final analyses.

The final questionnaire included 33 questions, only four of which were open-ended questions related to certain demographic details of the respondents. Some multiple-choice questions also had an option of 'others' for respondents to add their comments/choices beyond the choices provided. Not all questions were compulsory. A link to the survey was sent by email and to Indian physical therapy-based social media groups. The link remained active between February 18^th^ 2014 and May 5^th^ 2014. Reminder emails were sent every 3^rd^ week to non-responders. In the cover letter, respondents were informed that they were participating voluntarily and were free to withdraw at any time. They provided informed consent by clicking on the 'next' link in the survey. There were no financial benefits to the respondents of the survey.

The current evidence for the effectiveness of physical therapy interventions was collected using an electronic literature search performed in PubMed and Cochrane Database of Systematic Reviews. The search was limited to literature published in the previous fifteen years in English. Only systematic reviews and meta-analyses were considered. References of the articles were also searched for further relevant material. Based on the assessments of the review authors, the strength of evidence for the various interventions used for SIS was obtained. The current evidence regarding changes in kinematics and muscle activity associated with SIS was also searched to identify the correct strategies for exercise intervention for SIS patients.

Data were analyzed descriptively using SPSS software (SPSS Inc., Chicago, IL, USA), version 20.0. The proportions of physical therapists using different techniques were estimated and comparisons across gender, experience level, and training were made using chi square statistics. A p value of <0.05 was considered statistically significant. Furthermore, data were analyzed to see if the respondents' choice of therapy compared with their responses for etiology. For example, if a therapist claimed that abnormal kinematics or muscle strength imbalances are the most important cause for SIS, the choices of exercises that he/she made were subsequently analyzed.

The study protocol was approved by the Ethical Review Board of the Indian Spinal Injuries Center, New Delhi, India.

## Results

We received a total of 323 responses. Amongst these, 30 respondents were therapists practicing outside India and 82 were incomplete and hence excluded. The results were analyzed from the remaining 211 responses. The median time required to fill the survey was 19 minutes. More than half (55.5%) of the respondents had post-graduate specialization (usually with a duration of 2 to 3 years in India) (Table 1), most of which were in musculoskeletal/ orthopedic rehabilitation (45.5%) followed by sports therapy (15.7%), neurological rehabilitation (15.7%), cardiopulmonary rehabilitation (11.6%), and others (11.5%). Many also had training in manipulation therapy (55.5%), myofascial release techniques (30.8%), taping (27.0%), yoga (18.5%), osteopathy (16.1%), and acupuncture (12.8%). The years of clinical experience ranged between 0 to 30 years (mean ± SD = 4.9±5.1 years). 

**Table 1.** Demographics of the respondents of the survey.

**Demographic Parameter**	**Number of respondents** **(Total N=211) (%)**
**Gender**	121 males (57.3%)
**Age**	29.1±5.4 years
**Graduation**	
Undergraduate	86 (40.8%)
Graduate specialization	117 (55.5%)
Others	8 (3.8%)
**Year of graduation**	
1980-1989	3 (1.4%)
1990-1999	9 (4.3%)
2000-2009	99 (46.9%)
2010 onwards	100 (47.4%)
**Employment Setting**	
Academics	23 (10.9%)
Fitness Centers	4 (1.9%)
Hospital	46 (21.8%)
Inpatient care	3 (1.4%)
Other	8 (3.8%)
Private clinic	77 (36.5%)
Rehabilitation centers	11 (5.2%)
Research institute	2 (0.9%)
Sports association	3 (1.4%)
Student	31 (14.7%)
Wellness centers	3 (1.4%)

**Figure 1.**
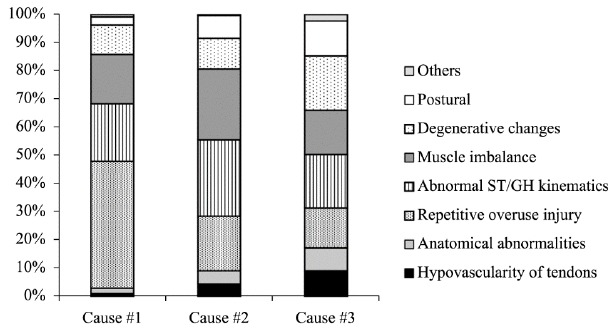
Relative percentages of respondents declaring their top three causes for SIS.

More than half (54.6%) responded that they treated 21-100 shoulder cases/year, and 13.5% responded that they treated >100 cases/year. Regarding etiology, the respondents ranked the three most significant causes of SIS as shown in Figure 1. In the subgroup that chose abnormal motion as the most significant cause, 

65.7% reported evaluation of posture in patients, 61.4% reported checking scapular dyskinesis in male patients and 28.6% in female patients. Overall, 83.9% of respondents reported using special tests to make their diagnosis of SIS.

Comparing the intervention choices made by physical therapists, 93.8% reported using exercise therapy and 92.4% reported using electrotherapeutic modalities (Table 2). Approximately three quarters reported using either capsular/muscle stretching or joint mobilization techniques. The reported use of taping techniques was relatively lower (68.5%) (Table 2). 

**Table 2.** Relative percentage of respondents using different intervention techniques for the management of SIS. The number who responded for each technique is included in parenthesis in column 1. The 'Yes' column depicts the percentage who claim confidence in the effectiveness of the use of the technique; 'Maybe' depicts the percentage who claim to use the technique with low or no confidence in its effectiveness; and 'No' depicts the percentage of those who either reported no knowledge of the technique or did not use the technique.

**Technique**	**Yes**	**Maybe**	**No**
Exercise Therapy (n=211)	76.3	17.5	6.2
Rotator cuff	81.8	16.2	2.0
Upper Trapezius	58.8	17.6	23.6
Middle Trapezius	65.2	23.8	11.0
Lower Trapezius	63.7	21.1	15.2
Serratus Anterior	73.1	11.0	15.9
Rhomboids	75.6	15.3	9.1
Latissimus Dorsi	51.8	27.1	21.1
Pectoralis Major	43.3	21.7	35.0
Pectoralis Minor	35.8	23.8	40.4
Biceps Brachii	45.3	22.4	32.3
Motor control of scapula	68.4	26.0	5.6
Closed kinetic exercises	45.5	37.4	17.1
Endurance exercises	51.0	26.3	22.7
Muscle relaxation	39.4	49.0	11.6
**Stretching Techniques (n=198)**	**55.6**	**21.2**	**23.2**
Upper Trapezius	79.4	10.7	9.9
Levator Scapulae	56.6	17.2	26.2
Rhomboids	39.3	17.0	43.7
Pectoralis Minor	61.9	16.1	22.0
Pectoralis Major	80.6	8.2	11.2
Latissimus Dorsi	38.7	28.8	32.5
Anterior Capsule	60.6	15.5	23.9
Posterior Capsule	53.4	17.3	29.3
Inferior Capsule	40.3	21.8	37.9
**Joint Mobilization Techniques (n=164)**	**57.3**	**18.3**	**24.4**
Glenohumeral	86.0	3.3	10.7
Scapulothoracic	70.5	9.5	20.0
Acromioclavicular	64.2	11.3	24.5
Thoracic	25.8	39.4	34.8
**Taping (n=165)**	**41.8**	**26.7**	**31.5**
Glenohumeral	64.4	15.8	19.8
Scapulothoracic	56.4	26.6	17.0
Correct Posture	71.4	17.3	11.3
**Therapeutic Modalities/Therapies (n=211)**	**63.5**	**28.9**	**7.6**
Cryotherapy	64.1	15.7	20.2
Superficial heating	60.9	16.5	22.6
Deep heating	46.2	21.4	32.4
Ultrasound	87.2	11.2	1.6
IFT	69.6	19.9	10.5
Muscle Stimulator	22.2	18.8	59.0
Laser Therapy	46.3	22.0	31.7
Extracorporeal Shockwave Therapy	8.3	12.8	78.9
Hydrotherapy	35.8	30.1	34.1

There were no significant differences in choices of exercise therapy and modalities across gender, experience levels (<5 years, 5-10 years, >10 years), and post-graduate training amongst the respondents.

Of all the respondents using exercise therapy, 80.8% claimed to design individualized exercise programs. However, no systematic patterns were identified. Almost all (98.0%) suggested that they strengthened the rotator cuff muscles (Table 2). Most respondents (>75%) reported strengthening of trapezius, serratus anterior, rhomboids, and latissimus dorsi. Over a fifth (23.6%) of respondents reported not prescribing resisted exercises or shoulder shrugs for strengthening upper trapezius. Two-thirds reported strengthening of pectoral muscles and biceps brachii. Almost all respondents (94.4%) suggested that they prescribed exercises to achieve movement control of scapula with varied levels of confidence. Amongst these, approximately four-fifths reported the use of closed kinetic positions and provision of visual (by mirrors) and/or verbal feedback. The percentage of respondents who suggested that they avoided exercises beyond 90 degrees early in rehabilitation was 71.8%. 

The number of respondents (61.9%) who declared confidence in the effectiveness of stretching the pectoralis minor was lesser than those who confidently prescribed stretching of the pectoralis major (80.6%) (Table 2). Thirty percent of the respondents declared that they used both strengthening and stretching exercises for pectoralis minor and 45% did so for upper trapezius. Though most respondents (88.4%) declared prescribing exercises for relaxation or reduction in muscle activity, only 40% claimed to have confidence in its effectiveness. Therapists usually reported targeting reduction of activity of upper trapezius, supraspinatus, or pectoralis minor. 

Respondents (62.1-76.1%) also reported stretching of different parts of the shoulder capsule (Table 2). Respondents who had additional training in mobilization or taping techniques were statistically more inclined to use them for SIS than those who were not trained (Chi square=5.55, df=1, p=0.018 and Chi square=9.48, df=1, p=0.002 respectively). 

The most preferred electrotherapeutic modalities were ultrasound and interferential therapy (Table 2). There was relatively no difference in the choice of superficial heat or cold. Respondents (80-95%) also reported teaching the patients to use ergonomic computer accessories and to avoid sleeping on the affected side, carrying heavy loads away from the body, carrying a shoulder strapped bag on the affected side, or raising the arm overhead. 

## Discussion

The results of the study indicate that Indian physical therapists partially follow the evidence for assessment and management of SIS. The choices of intervention were mostly appropriate to their reported beliefs for the etiology of the condition.

The understanding of the pathomechanics of SIS and evidence-based guidelines to manage the condition has evolved over the years^4^. Some of the potential reasons that may cause or aggravate the pathology are modifiable by physical therapeutic interventions. The study attempted to evaluate Indian physical therapists' level of awareness of current evidence-based practices.

A modifiable and often explored phenomenon associated with SIS is the change in kinematics and muscle function^23^^,^ 24. A physical therapeutic evaluation should therefore include assessment of muscle tightness, strength, and function beyond the special tests for SIS. Symptom alteration tests, such as scapular assistance^12^ and reposition tests^25^, are also recommended. In our study, we found that Indian physical therapists refrained from scapular examination, especially in females. This may be due to socio-cultural reasons. The authors believe that most women patients in India are not asked to undress adequately to correctly assess scapular dyskinesis. Therefore, therapists may fail to identify movement impairments to devise a targeted treatment approach. Furthermore, relatively fewer respondents reported (60%) assessment of posture. A dependence on special tests may help in a diagnosis, but does not guide exercise prescription^26^.

The survey result indicates that physical therapists claim to design individually tailored exercise regimes; however, they seem to prescribe generic and indiscriminate shoulder muscle strengthening and stretching exercises. Some respondents (~40%) reported that they did not differentiate between different parts of the trapezius and strengthened all parts of that muscle. However, the literature suggests that the upper trapezius causes scapular anterior tilt^23^, which reduces subacromial space, and therefore therapists should focus on reducing its activity by prescribing relaxation instead of strengthening exercises. Many respondents (91%) reported prescribing exercises for strengthening rhomboids, which are downward rotators of the scapula^23^. Reduced scapular upward rotation is seen in some patients with SIS^27^^,^ 28. Such patients should be prescribed middle/lower trapezius strengthening instead of rhomboid strengthening by performing scapular retraction in elevated arm positions (Prone T and Y exercises)^29^^,^ 30.

Respondents also reported prescribing exercises for strengthening internal rotators (65-78%) that are probably more prone to tightness than weakness. As terminal arm elevation requires upper trunk extension and glenohumeral external rotation, tightness in the pectoralis major and latissimus dorsi should not be overlooked. Stretching the pectoralis minor is especially important as it is attached to the coracoid process and its tightness is associated with decreased scapular posterior tilt^31^. The approximation of the rotator cuff tendons to the coracoacromial arch occurs much earlier than 90 degrees^10^, therefore avoiding arm elevation beyond 90 degrees (even without weight), as recommended by many of the respondents (72%), may not be a very purposeful strategy to prevent disease progression.

Over half (51%) of the respondents reported confidence in prescribing endurance exercises for patients with SIS. There is evidence that dyskinesis becomes evident after repetitive movement suggesting reduced fatigue resistance in scapular muscles^32^. Induced fatigue protocols also change scapular kinematics^33^^,^ 34. These findings suggest that an exercise program should incorporate targeted endurance exercises for shoulder muscles.

In accordance with the current literature^14^^,^ 16 ^,^ 19, more Indian physical therapists reported using exercises than glenohumeral mobilization techniques to manage the condition. Subjects with SIS usually have no limitation of movement, except internal rotation deficits in some posterior internal impingement patients^14^^,^ 35. There were a higher percentage of physical therapists who reported using mobilization techniques if they had special training for it. It is important to note that shoulder instability may lead to secondary SIS and hence capsular stretches and joint mobilization techniques should not be used indiscriminately^14^. Regarding the use of thoracic and acromioclavicular joint mobilization, there is inconclusive evidence for its effectiveness in the management of SIS^16^.

Similarly, there is lack of evidence supporting the use of certain electrotherapeutic modalities for the management of SIS. Among the respondents who rated either abnormal kinematics or muscle imbalance as the most important causes for the development of SIS, 90% reported using electrotherapeutic modalities. The very high response rate for the use of ultrasound in the current study is not supported by evidence^1^^,^ 16 ^,^ 19. Physical therapists should be cautious of using these modalities as the first or the only approach of management^16^.

The reasons for continued use of ultrasound or inconsistent exercise prescriptions may be due to lack of knowledge about the current evidence for SIS. This lack of awareness amongst Indian physical therapists may be due to difficulty in accessing scientific literature, lack of compulsory participation in continued education programs, and lack of timely revisions of curricula, which do not train therapists as movement analysts. The results of the current study helped to identify the gaps in current practices, which may help to formulate appropriate remedial strategies.

The results of the current study are comparable to a similar study with Dutch physical therapists^20^ on the relative preferences for various techniques and the higher use of manual mobilization by those with extra training for it. The authors of the study^20^ found that senior therapists tend to use these techniques more than younger ones. However, the same comparison was not possible in the current study because very few respondents were senior therapists (graduating before 1990).

A limitation of the current analyses is that the results are analyzed from what physical therapists claimed to be practicing. These self-reported responses may be biased for reasons of social desirability. To avoid this, the respondents were strongly advised in the cover letter to report what they actually practice. Secondly, as the design of the questionnaire was primarily multiple-choice questions, the respondents may have indiscriminately ticked the options. This would inflate the numbers of 'yes' or 'maybe' choices. However, if the questions were more open-ended, the analyses would be very difficult. The other limitation is that most of the respondents were recent graduates. However, the experience of therapists as deduced from the number of patients treated per year was fairly high. The response rates could not be estimated as the survey was sent using social media websites. Despite multiple reminders, the sample is not very large. This may be due to the absence of any financial benefits for participation for respondents. There were fewer overall responses for taping and mobilization technique as some respondents skipped those questions. This may indicate that they possibly did not use the technique; however, any attempts of such extrapolations are avoided in the study. Lastly, reliability testing was not done for the designed questionnaire.

The study identifies the strengths and weaknesses of current physical therapy practices in India, which may not be entirely generalizable to other countries. The findings should be interesting to practitioners and physical therapy educators as they may identify areas where awareness about current evidence needs to be enhanced. Future studies should include larger samples with more representation from senior therapists and compare therapy practices of different countries.

## Conclusion

For accurate assessment and targeted exercise prescription, it is crucial that therapists understand the pathomechanics of the disorder. The results of the study indicate that Indian physical therapists have a reasonable awareness of the current evidence for the management of SIS, but practices were only partially based on this evidence. The study helps to identify gaps in current physical therapy approaches to SIS in India and emphasizes the importance of continued education and timely revision of curricula.
